# Detection of Human Paragonimiasis by ELISA Using Recombinant *Paragonimus westermani* Cysteine Protease 7

**DOI:** 10.4269/ajtmh.22-0452

**Published:** 2023-05-30

**Authors:** Luana Gabriele Andrade-Gomes, María J. Zuniga, Gaby Dolz, Frank Solano-Campos

**Affiliations:** ^1^ Maestría en Enfermedades Tropicales, Posgrado Regional en Ciencias Veterinarias Tropicales, Universidad Nacional, Heredia, Costa Rica;; ^2^ Laboratorio de Zoonosis y Entomología, Programa de Medicina Poblacional, Escuela de Medicina Veterinaria, Universidad Nacional, Heredia, Costa Rica;; ^3^ Escuela de Ciencias Biológicas, Universidad Nacional, Heredia, Costa Rica

## Abstract

Paragonimiasis is an important but neglected foodborne trematodiasis caused by *Paragonimus mexicanus* in Costa Rica. Immunological techniques for diagnosing this parasitosis in humans do not exist in Central America. The objective of the present study was to use recombinant *Paragonimus westermani* cysteine protease 7 to standardize an ELISA for the detection of antibodies against *Paragonimus* spp. Human sera positive for *P. westermani*,* P. mexicanus*, or *Paragonimus* spp., human sera infected with other helminths, as well as sera of healthy humans without parasitic infections, were analyzed. The sensitivity of the ELISA was 92.9%, and the specificity was 91.9%. This report is the first to describe the development of an ELISA for the diagnosis of *Paragonimus* spp. in Costa Rica and Central America. Using this ELISA in the health system of Costa Rica is recommended to detect infections.

Paragonimiasis is a serious foodborne parasitosis caused by the consumption of raw or poorly cooked crustaceans containing metacercariae of the genus *Paragonimus*.^1^ It is also considered a neglected disease, causing an estimated of one million disability-adjusted life years worldwide.^2^ The species *Paragonimus mexicanus* is considered the most important pathogenic species in Latin America and causes lung but also ectopic infections, with the central nervous system being the most affected system.^3^ Misdiagnosis is common, and large lung lesions in humans are often interpreted as tuberculosis or malignant neoplasms.^4^^,^^5^

The definitive diagnosis of paragonimiasis is based on detection of eggs by microscopy in feces, sputum, aspirated fluids, or biopsies; however, the sensitivity is low (28–38%).^6^ Eggs are eliminated intermittently in the pulmonary form but not excreted at all in extrapulmonary cases, making the diagnosis impossible. For this reason, immunological assays are an important tool for the diagnosis of paragonimiasis.^7^^,^^8^ Immunoblotting and ELISA are the most frequently used methods, with the latter preferred for being simple and fast; however, the sensitivity and specificity of the technique depends on the type of antigen used.^9^

For the diagnosis of paragonimiasis, different antigens have been used: crude extracts of the parasite, excretory–secretory proteins of the parasite, and recombinant proteins (including cysteine proteases).^10^ The use of crude extracts of the parasite has the disadvantage of low specificity because they present cross-reactions with other parasites that share similar antigens.^11^ The use of excretory–secretory proteins, especially the cysteine proteases of the parasite, has been shown to increase diagnostic sensitivity and specificity.^12^ Because these proteins participate in a wide range of biological processes, their detection establishes an active infection, and they are also species-specific.^13^ To date, different recombinant cysteine proteases have been synthesized and used in the immunodiagnosis of paragonimiasis.^11^^,^^14^^–^^16^ Another advantage is that cysteine proteases are highly conserved, and most species of the genus *Paragonimus* have shared antigens, which facilitates the diagnosis of a species with the antigen of another species.^8^

In Costa Rica, paragonimiasis seems to be an underdiagnosed disease,^17^ especially because no immunological techniques are available to diagnose this parasitosis. Previous research on expression of CPs from *Paragonimus westermani* determined that three CPs (CP4, CP7, and CP8) where consistently expressed through the developmental stages of the parasite, from metacercaria to adult, while these recombinant antigens also showed the highest reactivity against serum samples from *P. mexicanus* and very low reactivity against other helminthic infections and normal controls.^15^ Therefore, we produced and tested these three recombinant antigens against serum samples from *P. mexicanus* via immunoblotting and identified recombinant *P. westermani* CP7 (rPwCP7) as the best candidate for detection of antibodies raised against *P. mexicanus*.^18^ The objective of the present study was to implement an ELISA using this antigen, which would allow early diagnosis of human paragonimiasis in Costa Rica.

The DNA sequence coding for the mature peptide of PwCP7 protein (DQ016550, 331-978 bp) was codon optimized and subcloned into the BamHI/HindIII sites of pET-28a (+) (Novagen, Pretoria, South Africa), in frame with the N-terminal His-tag by GenScript (Piscataway, NJ). Plasmid PwCP7-pET was transformed into chemically competent *Escherichia coli* BL21 Star (DE3) cells (Invitrogen, Carlsbad, CA). Cultures were grown in LB media at 37°C (225 rpm) until OD_600_ reached 0.8 and were induced with 1 mM IPTG for 3 h at 37°C. Cells were harvested by centrifugation at 5,000 × *g* for 10 min at 4°C and lysed with B-PER Complete (Pierce, Rockford, IL) containing protease and phosphatase inhibitor cocktail (Pierce).

Inclusion bodies (IBs) containing rPwCP7 were recovered from the bacterial lysate by centrifugation at 13,000 × *g* for 15 min at 10°C. IBs were washed in buffer 1 (2 M urea, 100 mM Tris, 5 mM ethylenediaminetetraacetic acid [EDTA], 5 mM dithiothreitol [DTT], and 1% Triton X-100) to remove impurities and soluble proteins associated with IBs,^19^ followed by buffer 2 (2 M urea, 100 mM Tris) to remove EDTA and DTT. IBs were solubilized in buffer 3 (100 mM Tris, 8 M urea) for 1 hour at room temperature. After centrifugation at 13,000 × *g* for 15 minutes at 10°C, the supernatant containing rPwCP7 was stored at −20°C.

The solubilized protein was purified by Ni-NTA spin columns (HisPur, Pierce) under denaturing conditions. Removal of urea and imidazole from the elutions was performed with Zeba spin desalting columns (Pierce) using 10 mM Tris HCl, pH 8 and 1 mM EDTA buffer, since these components, mainly urea, can affect antigen-antibody reactions.^20^ Protein purity was assessed using sodium dodecyl sulfate–polyacrylamide gel electrophoresis, and quantification was performed with Bradford assay kit (Pierce).

Western blot was carried out using the iBlot 2 system (Invitrogen) and the polyvinylidene difluoride membrane was blocked with I-Block (Applied Biosystems, Bedford, MA). rPwCP7 was detected with the His Tag antibody (GenScript), alkaline phosphatase-conjugate secondary antibody, and a chromogenic substrate (Invitrogen).

A total of 14 positive control sera samples from humans naturally infected with *P. westermani* (*n* = 8), and *P. mexicanus* (*n* = 6) were used that originated from Japan and were diagnosed by sequencing the eggs or the preadults and by ELISA. These sera were kindly provided by Dr. Hiromu Sugiyama, Department of Parasitology, National Institute of Infectious Diseases, Tokyo, Japan. As negative control sera, 133 serum samples from healthy individuals without parasitosis were used, these were collected in Costa Rica from volunteers without respiratory disease. Finally, human samples negative for *Paragonimus* but positive for other helminths (*Taenia solium* [*n* = 2], *Leishmania* sp. [*n* = 1], *Entamoeba histolytica* [*n* = 6], *Clonorchis sinensis* [*n* = 10], *Shistosoma japonicum* [*n* = 9], *Toxocara* sp. [*n* = 1], *Toxocara canis* [*n* = 1], *Fasciola* sp. [*n* = 3], and *Spirometra erinaceieuropaei* [*n* = 5]) were also donated by Dr. Sugiyama.

The ELISA was standardized as follows: polystyrene microtiter plates (Corning 2592, Kennebunk, ME) were used, which were sensitized with the rPwCP7 antigen, diluted in phosphate-buffered saline (PBS), and adsorbed at a concentration of 0.125 μg/well. After incubation for 16 hours at 4°C, the plate was washed three times (PBS, 0.05% Tween-20) and blocked for 16 hours at 4°C with 10% skim milk powder in PBS, and the blocking solution was removed without washing the plate. The serum was diluted at 1:100 in incubation solution (PBS, 0.05% Tween-20, and 5% skim milk) and incubated for 1 hour at 37°C. The wells were washed three times, and the conjugate (goat anti-human IgG-peroxidase, Sigma-Aldrich, Saint Louis, MO) diluted 1:40,000 in incubation solution was added, incubated for 30 minutes at 37°C, and washed again three times. The TMB (3,3′,5,5′-tetramethylbenzidine, Sigma-Aldrich) substrate was incubated at room temperature in the dark for 10 minutes and the reaction stopped with 50 μL of 2 M H_2_SO_4_. The absorbance reading was performed at 450 ηm with the Multiskan Ex (Thermo Scientific, Vantaa, Finland). One single positive control serum to *P. westermani* from Japan and one single negative healthy serum from Costa Rica were analyzed in quadruplicates throughout all the standardization experiments. Once the optical densities were obtained, the results were expressed as the average of the absorbance of the quadruplicates multiplied by 1,000, which was called ELISA units (EU). The cutoff point, sensitivity (Se) and specificity (Sp) was calculated using the receiver operating characteristic (ROC) curve with its respective area under curve (AUC).

The rPwCP7 protein showed strong bands of 27 kDa after purification in the E1-E5 elutions, indicating efficient and specific purification (Figure 1). The protein concentration obtained in elutions E1 and E2 after size exclusion chromatography was 200 μg/mL.

**Figure 1.**
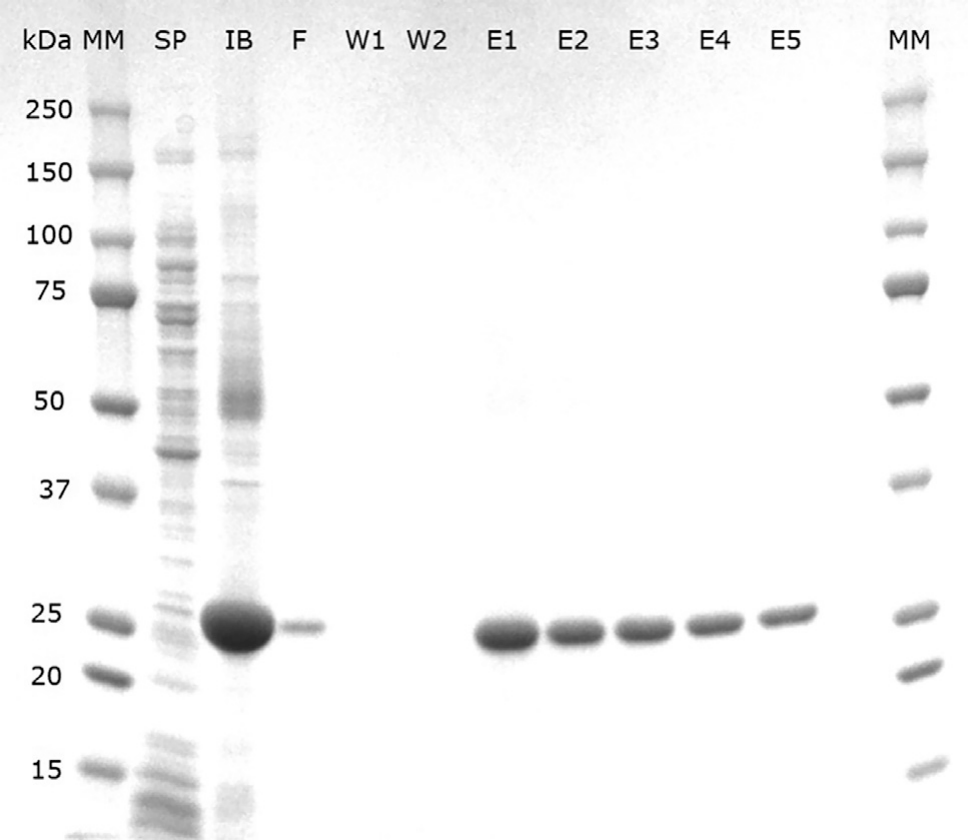
Sodium dodecyl sulfate–polyacrylamide gel electrophoresis and Coomassie stain for analysis of recombinant *P. westermani* cysteine protease 7 purification by metal chelate affinity chromatography. Lanes MM, precision plus protein kaleidoscope prestained standards; lane SP, soluble protein; lane IB, inclusion bodies solubilized in 8 M urea in Tris buffer; lane F, first flow-through; lanes W1 and W2, first and second washes; lanes E1–E5, first to fifth elutions.

During standardization of the ELISA, reproducibility of the positive and negative control sera over time and between plates was recorded, yielding EU between 600 and 680 and between 230 and 270, respectively. The 133 healthy negative control serum samples analyzed by ELISA yielded EU between 35 and 435. The cutoff point calculated by ROC curve (437 EU) showed high Se (92.9%) and Sp (91.9%). The performance of the immunoassay was high (AUC 0.96). Ten of 38 human samples negative for *Paragonimus* spp. but positive for *Fasciola* spp. (*n* = 1), *C. sinesis* (*n* = 3), *T. canis* (*n* = 1), *S. japonicum* (*n* = 3), and *S. erinaceieuropaei* (*n* = 2) reacted positive, and one *P. mexicanus* positive sera reacted negative in ELISA (Figure 2).

**Figure 2.**
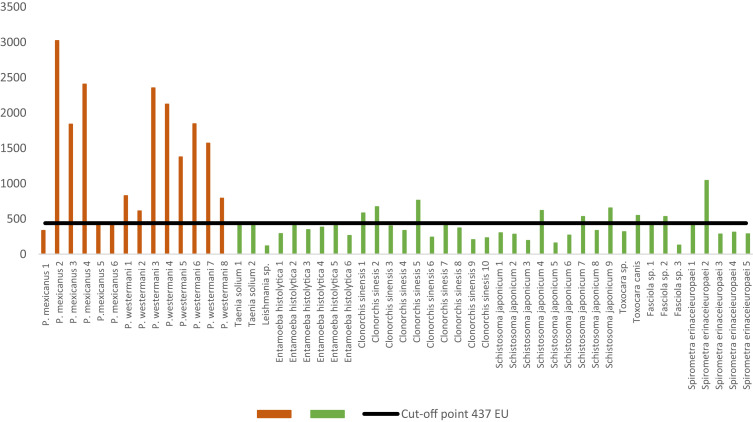
Results of the sera analyzed in the ELISA. Positive control sera are shown in red, and negative control sera but positive to other helminths are shown in green. EU = ELISA units.

Successful use of rPwCP7 as a diagnostic antigen has been reported in the literature^15^; thus, the rPwCP7 was produced and purified for use in ELISA to evaluate human sera positive for *Paragonimus* spp. The estimated yield of purified rPwCP7 was 400 μg from 300 mL of initial *E. coli* culture (1.3 mg/L). Because standardized ELISA uses 0.125 μg of protein per well, 3,200 serum samples can be evaluated with this amount of antigen. Another study conducted under similar conditions using the pET Expression System (Novagen) in *E. coli* BL21 (DE3) reported yields of 12.3 to 174.6 mg/L culture in the production of a papain-like cysteine protease from wheat, that also accumulated in insoluble inclusion bodies.^21^ The differences between the yield values obtained in the different studies may be related to the solubility of the proteins produced or to greater overexpression of the protein.^22^ Similarly, several recombinant cysteine proteases from *P. westermani*^13^ and *Paragonimus skrjabini*,^11^ expressed in *E. coli* BL21 (DE3), also aggregated in inclusion bodies but were soluble after purification and dialysis.

The Se and Sp of the ELISA were comparable to the values obtained by Pothong et al.,^8^ who used a recombinant protein of 35 kDa of *P. heterotremus* to detect antibodies in patients with paragonimiasis caused by *P. westermani* (Se 88.9% and Sp 95.5%). Although the proteins of different species of *Paragonimus* share many epitopes, limitations seem to exist when using the protein of one species as an antigen to diagnose another species,^8^ which may explain the low sensitivity obtained in the ELISA developed in this study. Another reason may be that the antibody titers in the sera analyzed were low.^11^ Cross-reactions were detected with five helminths, which agrees with the results obtained in 2015 by Ahn et al.^15^

This is the first report of the development of an ELISA using recombinant *P. westermani* cysteine protease 7 to diagnose paragonimiasis in Costa Rica and Central America. The implemented assay showed adequate sensitivity and specificity capable of detecting antibodies against *P. mexicanus*. For future studies, evaluating the use of a recombinant antigen of *P. mexicanus* is recommended to increase the sensitivity and specificity of the assay.

## References

[1] Sah R Khadka S, 2017. Case series of paragonimiasis from Nepal. Oxf Med Case Rep 11: 218–222. 10.1093/omcr/omx083 PMC5691872 29230303

[2] Morter R Adetifa I Antonio M Touray F Jong BC Gower CM Gehre F, 2018. Examining human paragonimiasis as a differential diagnosis to tuberculosis in The Gambia. BMC Res Notes 11: 31. 29334998 10.1186/s13104-018-3134-y PMC5769439

[3] Calvopina M Romero-Alvarez D Rendon M Takagi H Sugiyama H, 2018. *Hypolobocera guayaquilensis* (Decapoda: Pseudothelphusidae): a new crab intermediate host of *Paragonimus mexicanus* in Manabí Province, Ecuador. Korean J Parasitol 56: 189–194. 29742874 10.3347/kjp.2018.56.2.189 PMC5976022

[4] Cumberlidge N Rollinson D Vercruysse J Tchuenté LAT Webster B Clark PF, 2018. *Paragonimus* and paragonimiasis in West and Central Africa: unresolved questions. Parasitol 145: 1748–1757. 10.1017/S0031182018001439 30210013

[5] Kwon YS Lee HW Kim HJ, 2019. *Paragonimus westermani* infection manifesting as a pulmonary cavity and adrenal gland mass: a case report. J Infect Chemother 25: 200–203. 30213500 10.1016/j.jiac.2018.08.005

[6] Oey H , 2018. Whole-genome sequence of the oriental lung fluke *Paragonimus westermani.* Gigascience 8: 1–8. 10.1093/gigascience/giy146 PMC6329441 30520948

[7] Chen J , 2013. Cerebral paragonimiasis: a retrospective analysis of 89 cases. Clin Neurol Neurosurg 115: 546–551. 22795301 10.1016/j.clineuro.2012.06.025

[8] Pothong K Komalamisra C Kalambaheti T Watthanakulpanich D Yoshino TP Dekumyoy P, 2018. ELISA based on a recombinant *Paragonimus heterotremus* protein for serodiagnosis of human paragonimiasis in Thailand. Parasit Vectors 11: 322. 29843786 10.1186/s13071-018-2878-5 PMC5975669

[9] Narain K Dev KR Mahanta J, 2005. Development of enzyme-linked immunosorbent assay for serodiagnosis of human paragonimiasis. Indian J Med Res 121: 739–746. 16037618

[10] Blair D Toledo R Fried B Digenetic Trematodes, Vol 1154. Basel, Switzerland: Springer Nature Switzerland, 105–138.

[11] Yu S Zhang X Chen W Zheng H Ai G Ye N Wang Y, 2017. Development of an immunodiagnosis method using recombinant PsCP for detection of *Paragonimus skrjabini* infection in human. Parasitol Res 116: 377–385. 27796563 10.1007/s00436-016-5300-2

[12] Kim TY Joo IJ Kang SY Cho SY Kong Y Gan XX Sukomtason K Sukomtason K Hong SJ, 2002. Recombinant *Paragonimus westermani* yolk ferritin is a useful serodiagnostic antigen. J Infect Dis 185: 1373–1375. 12001061 10.1086/339880

[13] Yun D-H Chung J-Y Chung Y-B Bahk Y-Y Kang S-Y Kong Y Cho S-Y, 2000. Structural and immunological characteristics of a 28-kilodalton cruzipain-like cysteine protease of *Paragonimus westermani* expressed in the definitive host stage. Clin Diagn Lab Immunol 7: 932–939. 11063501 10.1128/cdli.7.6.932-939.2000 PMC95988

[14] Yoonuan T Nuamtanong S Dekumyoy P Phuphisut O Adisakwattana P, 2016. Molecular and immunological characterization of cathepsin L-like cysteine protease of *Paragonimus pseudoheterotremus.* Parasitol Res 115: 4457–4470. 27562899 10.1007/s00436-016-5232-x

[15] Ahn C Na B Chung D Kima J Kim J Konga Y, 2015. Expression characteristics and specific antibody reactivity of diverse cathepsin F members of *Paragonimus westermani.* Parasitol Int 64: 37–42. 25284814 10.1016/j.parint.2014.09.012

[16] Curtis KC Fischer K Choi YJ Mitreva M Weil GJ Fischer PU, 2021. Characterization and localization of antigens for serodiagnosis of human paragonimiasis. Parasitol Res 120: 535–545. 33415393 10.1007/s00436-020-06990-z PMC7854406

[17] Hernández-Chea RD, 2016. *Caracterización morfológica y molecular de* Paragonimus mexicanus *y* Paragonimus caliensis *en cangrejos de agua dulce de Costa Rica*. MS Thesis, Universidad Nacional, Heredia, Costa Rica.

[18] Andrade-Gomes LG Solano-Campos F Dolz G, 2017. Evaluación de proteínas recombinantes PwCP4, PwCP7 y PwCP9 usando sueros de ratas Wistar infectadas experimentalmente con *Paragonimus mexicanus.* Cienc Vet (Heredia) 36: 44

[19] Palmer I Wingfield PT, 2012. Preparation and extraction of insoluble (inclusion‐body) proteins from *Escherichia coli.* Curr Protoc Protein Sci, doi: 10.1002/0471140864.ps0603s70. PMC3809847 23151747

[20] Bata JE Gyenes L Sehon AH, 1964. The effect of urea of antibody-antigen reactions. Immunochemistry 1: 289–293. 14250782 10.1016/0019-2791(64)90029-1

[21] Gorokhovets NV , 2017. Rational design of recombinant papain-like cysteine protease: Optimal domain structure and expression conditions for wheat-derived enzyme triticain-α. Int J Mol Sci 18: 1395. 28661426 10.3390/ijms18071395 PMC5535888

[22] Zhu S Gong C Ren L Li X Song D Zheng G, 2013. A simple and effective strategy for solving the problem of inclusion bodies in recombinant protein technology: His-tag deletions enhance soluble expression. Appl Microbiol Biotechnol 97: 837–845. 23250226 10.1007/s00253-012-4630-y

